# Use of Os-Artificial

**Published:** 1869-10

**Authors:** 


### Editorial.

### USE OF OS-ARTIFICIAL.

THE object of the faithful Dentist will ever be to enlarge the sphere of his usefulness, and to accomplish this he must bring to his aid whatever of methods and materials may be made available. The use of os-artificial as a covering for exposed pulps has been fully described by some who early employed it for this purpose. The method proposed in this description, and since employed by many in the profession, is simply the covering the orifice of pulp exposure by the os-artificial, bringing it in contact with the exposed pulp. In many cases the pulp is so susceptible to the action of the zinc chloride, as to take on inflammation, become painful and devitalized, following which will be the usual train of circumstances attending such conditions. Such a result may ordinarily be averted by preventing the contact of the escharotic. This may be accomplished by either of two or three simple methods, first, by placing carefully over the orifice of exposure a small thin piece of Hill's stopping, or even gutta percha softened by heat, so as thoroughly to protect the pulp from the contact of the zinc chloride.

A more perfect covering, and one that will certainly occupy all space, may be made by a coating of collodin, or a thick solution of gutta percha and chloroform, this is preferable to the former. A thick covering of the solution is placed over and around the orifice of exposure, and the chloroform may be almost instantly evaporated by throwing into the cavity a jet of warm air. If there is an excess of the covering it may be trimmed off. The cavity after being perfectly dried by the warm air jet is ready for the os-artificial, which should have as great consistence as the proper manipulation will admit. The cavity in all cases may be completely filled, and if a gold filling is to be made, the os-artificial will cut away to the proper point.

Instead of using the solution for a covering, as suggested, a small pledget of cotton moistened with carbolic acid, or any other agent indicated may be placed on the orifice of exposure, and then the cavity filled as before. Another method of protecting the exposed pulp from the action of the zinc chloride is cauterization by nitric acid, after which the os-artificial may be directly applied, and will seldom, if ever cause the slightest pain. Os-artificial can not be relied upon for permanent fillings, except to some extent in certain cases. We regard it, however, as invaluable for the purpose already mentioned. The extent to which the os-artificial should be excavated for filling with gold, will be determined by the size and depth of the original cavity. In all cases, however, sufficient excavations should be made to admit of a firmly attached, and well adapted filling.

In a shallow cavity there will be a mere lamina of the primary filling, while in those of greater depth, one-third to two-thirds of the cavity may be filled by it. Os-artificial of the best quality only, such as will become very hard, should be used for this purpose. It then affords an excellent foundation for gold filling. We doubt not, that with proper care, as good gold fillings may be made upon a base of this kind, as upon dentine itself, providing, always, that the walls of the cavity are of dentine and are sufficient to guarantee a good support. The os-artificial should, in no case, extend to the orifice of the cavity.

In teeth the pulps of which are gone, with a large cavity, pulp chambers and canals of roots, all to be filled, may be treated upon the general plan we have indicated, with an assurance of success equal to any other.

After the most careful preparation of the whole case in the usual manner, the canals in the roots should be filled with gold, then fill the pulp chamber and decayed cavity with the os-artificial, then excavate this to the proper extent and fill with gold.

There are two or three advantages accruing from this method of operating, especially in extreme cases, that are quite apparent upon the mere statement, and the first is where there are living pulps the ease and facility thus afforded of introducing gold fillings without a possibility of the slightest injury to the pulp. Another is the entire protection of the pulp from thermal changes, thus placing it in as nearly its original condition as it seems possible.

Again, in those large and very prolonged operations, as heretofore performed with gold only, involving excessive fatigue to both patient and operator, the work, by the method we have suggested becomes very much less laborious and fatiguing to all concerned.

It may be said, this is a matter of no consequence; but it is a matter of very great importance when we consider that some of the very best men in our profession have become broken down and worn out by the excessive labor and fatigue involved in prolonged and tedious operations. Our experience in the methods indicated above runs through about four years, with no other indications than those of the most desirable character. T.

